# High quality metabolomic data for *Chlamydomonas reinhardtii*

**DOI:** 10.1186/1746-4811-4-7

**Published:** 2008-04-28

**Authors:** Do Yup Lee, Oliver Fiehn

**Affiliations:** 1 University of California Davis, Genome Center, Davis, CA 95616, USA

## Abstract

The green eukaryote alga *Chlamydomonas reinhardtii *is a unicellular model to study control of metabolism in a photosynthetic organism. We here present method improvements for metabolite profiling based on GC-TOF mass spectrometry focusing on three parameters: quenching and cell disruption, extract solvent composition and metabolite annotation. These improvements facilitate using smaller cell numbers and hence, smaller culture volumes which enable faster and more precise sampling techniques that eventually lead to a higher number of samples that can be processed, e.g. for time course experiments. Quenching of metabolism was achieved by mixing 1 ml of culture to 1 ml of -70°C cold 70% methanol. After centrifugation, cells were lyophilized and disrupted by milling using 2-6E6 lyophilized cells, around 500-fold less than previously reported. Glass beads were compared to metal balls for milling, and five different extraction solvents were tested. Additionally, all peaks were annotated in an automated way using the GC-TOF database BinBase instead of manual investigation of a single reference chromatogram. Median precision of analysis was used to decide for the eventual procedure which was applied to a proof-of-principle study of time dependent changes of metabolism under standard conditions.

## Introduction

*Chlamydomonas reinhardtii *is a model system for photosynthetic organisms [1] including studies on metabolism [2-4]. It has been studied since long as a particularly sturdy organism that can be genetically modified in multiple ways and for which community resources are available including mutant stock centers and a fully sequenced genome. *Chlamydomonas *may also be used for studying the response to availability of macronutrients e.g. phosphate, sulfur, carbon, and nitrogen [5] which was extended to broad profiling of responses of gene expression or metabolite levels [6,7]. The focus of such studies is to understand the complexity of regulatory circuits and reorganization of cellular modules in response to suboptimal conditions which may then lead to insights that could potentially be extended to vascular plants.

Metabolites can be regarded as the ultimate output of the cellular machinery. Therefore, comprehensive metabolic phenotyping may help to unravel subtle stages of cellular reorganization if highly accurate quantifications can be achieved. Analytical methods have to be constantly improved in order to achieve this aim. One of the main concerns for developing analytical methods for quantifying microbial metabolites is to prevent undesirable changes of internal metabolites during the period of harvesting. The aim is to stop any metabolic activity as fast as possible without altering the internal metabolic signature. Yeast may be regarded as good proxy for *Chlamydomonas *with respect to sample preparation as both are eukaryotic organisms exerting comparatively sturdy cell walls, unlike bacterial models which are known to be more easily disrupted by physicochemical methods. Yeast metabolism has been preferably quenched by cold methanol treatments [8]. Nevertheless, even mild quenching methods unavoidably may lead to some degree of metabolite leakage by weakening cell walls. Consequently minimal concentrations of methanol and/or centrifugation times were tested, as well as alternative methods such as rapid filtration, for bacteria [9,10]. Other studies have focused on the optimization of extraction methods to obtain a comprehensive overview of metabolism despite the known diverse physicochemical nature of metabolite structures. Different cellular disruption methods were investigated for mycobacteria [11] and extraction efficacies were optimized for metabolite profiling of a variety of matrices such as Arabidopsis tissues [12], E. coli [13,14]), yeast cells [15] or blood plasma [16]; each yielding quite different protocols. These efforts document that sample preparation methods have to be carefully worked out and cannot be transferred from one field of application to the other without in-depth validation.

We have previously reported an initial protocol for metabolite profiling of *Chlamydomonas *strains and demonstrated that losses of internal metabolites were minimal during the quenching and centrifugations steps [7]. However, the final method proved to be very labor intensive and unpractical for higher number of samples because it involved grinding samples with mortar and pestle and high volumes of quenching solutions. We here report to advance this protocol by focusing on high reproducibility of quantitative metabolite profiling results by standardizing algae growth, by miniaturizing the sample volumes and by altering the cell disruption method, and the quenching and extraction procedure. In addition, we here report improved data acquisition and data processing steps that were not used before for Chlamydomonas profiling.

## Materials and methods

Experimental details are given according to the minimal reporting standards laid out for microbial biology context information, chemical analysis and data processing, as published by the Metabolomics Standards Initiative.

### Growth conditions

The *Chlamydomonas reinhardtii *strain CC125 was used for all studies. The strain was cultivated in TAP medium [17] at 23°C under constant illumination with cool-white fluorescent bulbs at a fluence rate of 70 μmol m^-2 ^s^-1 ^and with continuous shaking. Cryopreserved stocks [18] were used to inoculate a starter culture, which was harvested at late log-phase and used to inoculate a new culture at a starting density of 5 × 10^5 ^cells/mL. All cell numbers were counted with a Coulter automated cell counter. After 48 h, cells were harvested by centrifugation, washed twice with sterile 20 mM TRIS pH 7.0, supplied with 300 mM CaCl2, 400 mM MgCl_2_, and 7 mM KCl, and resuspended at a starting density of 2.5 × 10^6 ^cells/mL in TRIS-buffered media under standard growth conditions [19] using 20 mL total volume in 125 mL beakers. 1 mL of cell culture was harvested at 1 h, 4 h, 10 h, and 22 h.

### Preparation of cell extracts

At the incubation site 1 mL cell suspensions were injected into 1 mL of -70°C cold quenching solution composed of 70 % methanol in water using a thermo block above dry ice. Centrifuge tubes containing the solution during harvest were cooled in a pre-chilled cooling box to keep sample temperature below -20°C. Cells were collected by centrifugation at 16,100 rcf for 2 min with the centrifuge and rotor cooled at -20°C. Supernatant was decanted and residual liquid carefully removed. The pellet was flash frozen in liquid nitrogen and lyophilized at -50°C in a 2 mL round bottom Eppendorf tube.

Lyophilized cells were disrupted using the ball mill MM 301 (Retsch GmbH & Co., Germany) in two variants for 3 minutes: (a) using 0.5 ml of glass beads (500 μm i.d., Sigma, St.Louis MO) concomitant with 0.5 ml extraction solvent and (b) using a single 5 mm i.d. steel ball, followed by the addition of 0.5 mL extraction solvent and vortexing. After 2 min centrifugation at 16,100 rcf, the supernatants were removed (200 μl for the glass bead method, 350 μl for the steel ball method) followed by a secondary extraction step using an additional 800 μl extraction solvent, centrifugation and adding the supernatant (700 μl for the glass bead method) to the first aliquot. Hence, for both methods the same fraction of extraction solvent was used (70% of total extraction volume).

Five different extraction solvent systems were tested: methanol:chloroform:water (5:2:2) as published for plant organs [12,20], methanol:isopropanol:water (5:2:2), 100% methanol [15], acetonitrile:isopropanol:water (5:2:2) as published for blood plasma extractions [21] and methanol:chloroform:water (10:3:1) which was previously found to be most suitable for *Chlamydomonas *profiling [7]. Solvent ratios are given as volumetric measures. All solvents were degassed by directing a gentle stream of nitrogen through the solvent for 5 min and were used pre-chilled to -20°C prior to extraction. HPLC grade methanol, isopropanol, LC grade acetonitrile and purified grade chloroform were supplied by Mallinckrodt Baker Inc. (Phillipsburg. NJ, USA). Pure water was supplied by a MilliQ gradient A10 unit (Millipore Corporation, Billerica, MA, USA) with a residual total organic carbon content of less than 5 ppb.

Extracts were concentrated to dryness in a vacuum concentrator. Dried extracts were kept at -80°C for up to 4 weeks before derivatization and analysis by GC-TOF mass spectrometry.

#### Data acquisition by GC-TOF mass spectrometry

##### Sample preparation

A mixture of internal retention index markers was prepared using fatty acid methyl esters of C8, C9, C10, C12, C14, C16, C18, C20, C22, C24, C26, C28 and C30 linear chain length, dissolved in chloroform at a concentration of 0.8 mg/ml (C8-C16) and 0.4 mg/ml (C18-C30). 2 μl of this RI mixture were added to the dried extracts. 5 μl of a solution of 20 mg/ml of 98% pure methoxyamine hydrochloride (CAS No. 593-56-6, Sigma, St.Louis MO) in pyridine (silylation grade, Pierce, Rockford IL) was added and shaken at 30°C for 90 min to protect aldehyde and ketone groups. 45 μl of MSTFA.1%TMCS (1 ml bottles, Pierce, Rockford IL) was added for trimethylsilylation of acidic protons and shaken at 37°C for 30 min. Reaction mixtures were transferred to 2 ml clear glass autosampler vials with microinserts (Agilent, Santa Clara CA) and closed by 11 mm T/S/T crimp caps (MicroLiter, Suwanee GA).

##### Auto injector

A Gerstel automatic liner exchange system with multipurpose sample MPS2 dual rail and two derivatization stations was used in conjunction with a Gerstel CIS cold injection system (Gerstel, Muehlheim, Germany). For every 10 samples, a fresh multibaffled liner was inserted (Gerstel #011711-010-00) using the Maestro1 Gerstel software vs. 1.1.4.18. Before and after each injection, the 10 μl injection syringe was washed three times with 10 μl ethyl acetate. 1 μl sample was filled using 39 mm vial penetration at 1 μl/s fill speed, injecting 0.5 μl at 10 μl/s injection speed at initial 50°C which was ramped by 12°C/s to final 250°C and hold for 3 minutes. The injector was operated in splitless mode, opening the split vent after 25 s.

##### Chromatography instrument

An Agilent 6890 gas chromatograph (Santa Clara CA) was controlled by the Leco ChromaTOF software vs. 2.32 (St. Joseph MI).

##### Separation column

A 30 m long, 0.25 mm i.d. Rtx-5Sil MS column with 0.25 μm 95% dimethyl 5% diphenyl polysiloxane film and additional 10 m integrated guard column was used (Restek, Bellefonte PA).

##### Separation parameters

99.9999% pure Helium with built-in purifier (Airgas, Radnor PA) was set at constant flow of 1 ml/min. The oven temperature was held constant at 50°C for 1 min and then ramped at 20°C/min to 330°C at which it was held constant for 5 min.

##### Mass spectrometer

A Leco Pegasus IV time of flight mass spectrometer was controlled by the Leco ChromaTOF software vs. 2.32 (St. Joseph, MI) and operated by Do Yup Lee.

##### Sample introduction

The transfer line temperature between gas chromatograph and mass spectrometer was set to 280°C.

##### Ionization

Electron impact ionization at 70V was employed with an ion source temperature of 250°C.

##### Data acquisition

After 290 s solvent delay, filament 1 was turned on and mass spectra were acquired at mass resolving power R = 600 from m/z 85–500 at 10 spectra s^-1 ^and 1800 V detector voltage without turning on the mass defect option. Recording ended after 1200 s. The instrument performed autotuning for mass calibration using FC43 (Perfluorotributylamine) before starting analysis sequences.

#### Data pre-processing

##### File formats

Files were preprocessed directly after data acquisition and stored as ChromaTOF-specific *.peg files, as generic *.txt result files and additionally as generic ANDI MS *.cdf files.

##### Pre-Processing details

ChromaTOF vs. 2.32 was used for data preprocessing without smoothing, 3 s peak width, baseline subtraction just above the noise level, and automatic mass spectral deconvolution and peak detection at signal/noise levels of 10:1 throughout the chromatogram. For each peak, the apex masses and the complete spectrum with absolute intensities were exported, along with retention time, peak purity, noise, signal/noise ratio, unique ion and unique ion signal/noise ratio. Further metadata were also exported but are not yet used in the BinBase algorithm [22]. Result *.txt files were exported to a data server with absolute spectra intensities and further processed by the BinBase algorithm. This algorithm used the settings: validity of chromatogram (<10 peaks with intensity >10^7 counts s^-1^), unbiased retention index marker detection (MS similarity>800, validity of intensity range for high m/z marker ions), retention index calculation by 5^th ^order polynomial regression. Spectra were cut to 5% base peak abundance and matched to database entries from most to least abundant spectra using the following matching filters: retention index window ± 2,000 units (equivalent to about ± 2 s retention time), validation of unique ions and apex masses (unique ion must be included in apexing masses and present at >3% of base peak abundance), mass spectrum similarity must fit criteria dependent on peak purity and signal/noise ratios and a final isomer filter (if two closely related isomer spectra were found, the spectra with the closer proximity of the database RI value was taken and the alternative isomer spectrum was re-assessed). Failed spectra were automatically entered as new database entries if s/n >25, purity <1.0 and presence in the biological study design class was >80%. All thresholds reflect settings for ChromaTOF vs. 2.32. BinBase automatically recognizes data processed by ChromaTOF vs. 3.25 but thresholds for spectra quality are not yet validated. Quantification was reported as peak height from the absolute ion intensity using the unique ion as default quantification mass, unless a different quantification ion was manually set in the BinBase administration software Bellerophon. A quantification report table was produced for all database entries that were positively detected in more than 80% of the samples of a study design class (as defined in the SetupX database [23]). This procedure results in 10–30% missing values which could be caused by true negatives (compounds that were below detection limit in a specific sample) or false negatives (compounds that were present in a specific sample but that did not match quality criteria in the BinBase algorithm. A subsequent post-processing module was employed to automatically replace missing values from the *.cdf files using the open access mzmine software [24] under the following parameters: for each positively detected spectrum, the average retention time was calculated and intensities of the quantification ions were subtracted by the lowest background intensity in a retention time region of ± 5 s. The resulting report table did not comprise any missing values, but replaced values were labeled as 'low confidence' by color coding.

##### Data transformation

Result files were transformed by calculating the sum intensities of all structurally identified compounds for each sample and subsequently dividing all data associated with a sample by the corresponding metabolite sum. The resulting data were multiplied by a constant factor for convenience of obtaining values without decimals. Intensities of identified metabolites with more than one peak (e.g. for the syn- and anti-forms of methoximated reducing sugars) were summed to only one value in the transformed data set. The original non-transformed data set was retained.

##### Statistics

Statistical analyses were performed on all continuous variables using the Statistica software vs. 7.1 (StatSoft, Tulsa OK). Univariate statistics for multiple study design classes was performed by breakdown and one-way ANOVA. F-statistics and p-values were generated for all metabolites. Data distributions were displayed by box-whisker plots, giving the arithmetic mean value for each category, the standard error as box and whiskers for 1.96 times the category standard error to indicate the 95% confidence intervals, assuming normal distributions. Multivariate statistics was performed by unsupervised principal component analysis (PCA) to obtain a general overview of variance of metabolic phenotypes in the study, by entering metabolite values without study class assignments. In addition, supervised partial least square statistics were performed which requires information about the assigned study classes. Three plots were obtained for each PCA and PLS model: (i) the scree-plot for the Eigenvalues of the correlation or covariance matrix. This is considered as a simple quality check and should have a steep descent with increasing number of Eigenvalues. (ii) Secondly, 2D score scatter plots were generated for at least the first three dimensionless principal components. 3D plots are generated for better distinguishing metabolic phenotypes. (iii) Thirdly, loadings plots were generated for each vector in PCA or PLS showing the impact of variables on formation of vectors. Metabolites near the coordinate center had no separation power; conversely, variables far away from the coordinate center were important for building PCA and PLS models. Variables that are located close to each other are strongly correlated.

#### Quality controls

Daily quality controls were used. These comprised two method blanks (involving all the reagents and equipment used to control for laboratory contamination) and four calibration curve samples spanning one order of dynamic range and consisting of 31 pure reference compounds. 0.5 μl injection volumes and split ratio of 1/5 were used. Intervention limits were established and laid out in a Standard Operating Procedure to ensure the basic validation of the instrument for metabolite profiling.

#### Metabolite identifications

For this study, only two of the four possible MSI-categories of metabolite identifications were used, 'identified compounds' and 'unknown compounds'. Both categories were unambiguously assigned by the BinBase identifier numbers, using retention index and mass spectrum as the two most important identification criteria. Additional confidence criteria were given by mass spectral metadata, using the combination of unique ions, apex ions, peak purity and signal/noise ratios as given in data preprocessing. Every database entry in BinBase is routinely matched against the Fiehn mass spectral library that had been generated using identical instrument parameters as given above. Currently, the Fiehn library hosts 713 unique metabolites with a total of 1,197 unique spectra. BinBase entries were named manually by matching mass spectra and retention index. For named BinBase compounds, PubChem numbers and KEGG identifiers were added. In addition, all reported compounds (identified and unknown metabolites) are reported by the quantification ion and the full mass spectrum which is encoded as string.

## Results and Discussion

### Chlamydomonas cultures can be grown in a highly reproducible manner

**Figure 1:**
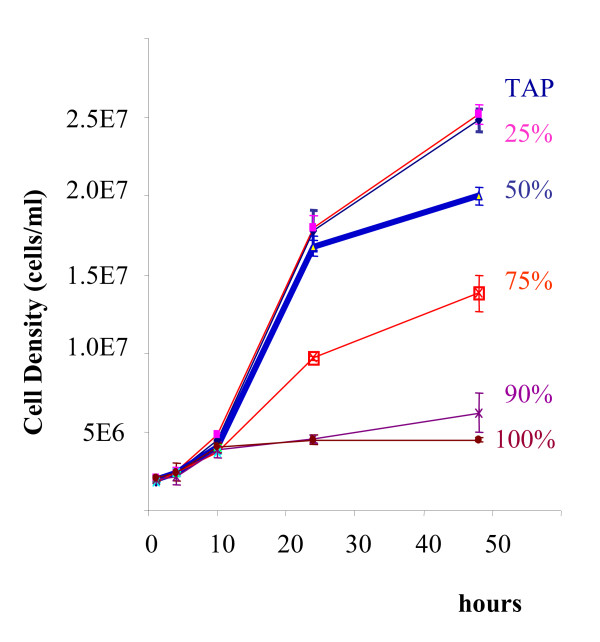
Precision of *C. reinhardtii *growth rates in three independent replicate studies on the impact of ammonium depletion using standard TAP medium and 25–100% ammonium depleted TAP medium.

In order to establish the reproducibility of growth rates at physiologically different conditions, *Chlamydomonas reinhardtii *cell cultures were grown in three completely independent replicate studies. Each series was started roughly one week apart, using fresh starting and inoculating cultures which were grown under standard conditions with TRIS-acetate-phosphate (TAP) media and compared to TAP media that had 25, 50, 75, 90 and 100% reduced ammonium concentrations. Figure 1 demonstrates that growth rates and absolute cell numbers were highly reproducible if both media and starting cell numbers were scrupulously controlled. The high precision of independent experiments indicates that *Chlamydomonas *can be used for broad scale comparisons of physiological, biochemical and genetic perturbations and their corresponding impacts on metabolic responses, even, if studies are set up in separate batches. In order to prove this comparability in each case, it is highly advisable to refer to one standard condition that is identical in all cases.

### Chlamydomonas GC-MS profiles encompass free fatty acids if special injection systems are used

**Figure 2:**
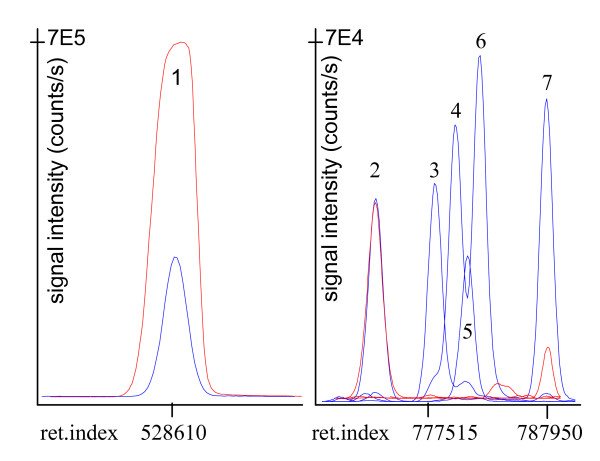
Comparison of *C. reinhardtii *GC-TOF metabolite profiles by polar fractionation and sample overloading (red ion traces) versus injecting complete extracts (blue) with optimized extraction and injection methods. Left panel, peak #1 glutamate m/z 246. Right panel, signal intensity normalized to peak #2 (unidentified sugar phosphate). Peak #3 linoleic acid m/z 337, #4 and #6 oleic and elaidic acid m/z 339, # 5 linolenic acid m/z 335, #7 stearic acid m/z 341.

For optimizing quenching, grinding and extraction conditions for *Chlamydomonas *metabolite profiling, we have exclusively used cultures grown under standard conditions using TAP media. First, the number of cells were reduced to 2.5 × 10^6 cells per sample for sample preparation compared to a method that reportedly frequently used more than 10^9 cells [7]. In addition, the extraction method was modified such that no lipid/polar fractionation was carried out. Instead, injections into the GC-TOF mass spectrometer were performed using automatic liner exchange and multibaffled liners under cold injection conditions [25]. These improvements greatly limited matrix effects and cross-contamination by semi-volatile breakdown artifacts even when non-fractionated extracts were injected. In methods published before, glass wool liners were used to prevent breakthrough of contaminants onto the GC columns; however, inevitably these liners get increasingly contaminated as evidenced by dark deposits indicating slow pyrolysis processes. Using very frequent liner exchanges, however, full extracts can be injected that facilitate the quantitative analysis of free fatty acids and sterols along with polar metabolites such as sugar phosphates and amino acids. In figure 2, results are compared using a representative sample for both methods. It becomes evident that free fatty acids such as linoleic, linolenic, oleic and stearic acid are present in high concentrations in full *Chlamydomonas *extracts [26], much higher than, for example, in leaf extracts of *Arabidopsis thaliana*. Conversely, if extracts undergo polar/lipid fractionations, no unsaturated fatty acids are detectable (fig. 2). We have further investigated potential breakdown of membrane lipids in full extracts by comparing the procedure as given in the 'methods' section to results using a secondary clean-up step. In this secondary step, dried extracts were resuspended with 50% acetonitrile and water. This step proved efficient in removing complex polar lipids in exemplary LC-MS analyses. When comparing results in GC-TOF profiles, we did not find levels of free fatty acids to be significantly lower using such a clean-up step (data not shown) and we conclude that the fatty acid levels in our Chlamydomonas profiles indeed reflect endogenous metabolic concentrations and are not caused by potential breakdown of membrane lipids. Further evaluations confirmed that no further interferences with the quantification of other metabolome constituents (e.g. amino acids) were detected and it was concluded that a secondary clean-up step is unnecessary. Further control experiments proved that repetitive blank runs after full extract injections did not show increased levels of fatty acid peaks if cold injection and liner exchange is used which confirmed that the observed levels of fatty acids were not due to potential carry-over effects. This observation is radically different from hot injections into classic split/splitless glass wool-containing liners for which increasing intensities of artifact formation of fatty acid peaks are found if full extracts are injected. These findings indicate that it is possible and necessary to target free fatty acids in Chlamydomonas in order to get more comprehensive overview of metabolic changes. In addition, overloading of chromatograms is observed if too high cell numbers are used for extraction. Figure 2 compares peak shapes for derivatized glutamate using the previously published method and the optimized injection conditions. Mass spectrometers suffer from limited dynamic range of quantification, specifically time-of-flight instruments. If ion detection reaches detector saturation as observed for glutamate levels when a very high number of Chlamydomonas cells is used, peak heights quickly enter a non-linear response of signal intensity versus injected amount of compounds. Such sample overloading renders quantitative assessments hardly reproducible. If chromatograms were to be investigated manually, one could select low abundance mass spectral ions which suffer less from detector saturation in order to improve the quantification accuracy. However, it is hardly possible to run large comparative studies using manual investigations of chromatograms. The automated annotation of GC-TOF profiles using the BinBase database is restricted to peak height comparisons [22] and therefore, employing lower number of cells and less amounts of extracts yield more precise quantitative data than manual investigations and high numbers of Chlamydomonas cells. Inevitably, however, comparatively low abundant metabolites may not be detectable if too low injection amounts are used. The dilemma of overloaded peaks on the one hand and not detectable (low abundant) metabolites on the other hand can only be resolved if mass spectrometers with larger dynamic ranges are employed, or if multiple fractionations and data acquisitions are used that target at different chemical classes of the metabolome.

### Method improvements for quenching Chlamydomonas cell cultures

We have utilized the previously published method [7], here called the benchmark protocol, for step wise improvements of the sample preparation process. Rapid stop of metabolism by inactivating enzymes as fast and as mild as possible are mandatory to retain a valid snapshot of metabolism at the point of harvest [15,27]. Accordingly, cells were rapidly quenched to temperatures below -20°C at harvest, although, inevitably, certain enzymes with very high turnover rates may still remain active long enough to alter metabolite ratios, e.g. for ADP/ATP. GC-TOF mass spectrometry cannot determine ADP and ATP and hence, metabolite profiling techniques and optimizations used here aimed only at the detectable compounds.

Methanol concentrations in the quenching solution were carefully increased in order to facilitate lower chill temperatures of the quenching solutions and correspondingly, lower volumes that were required for quenching. At a methanol/water ratio of 70:30, the quenching solution could be cooled to -70°C without phase separation. When this solution was used in 1:1 ratio with room temperature cell cultures under rapid vortexing, a final temperature of -20°C was measured and a final methanol concentration of 35%.

This final 35% methanol concentration was higher than used in the benchmark protocol (26%) and therefore, a higher amount of leakage could potentially occur. We have here determined leakage by analyzing intracellular peak intensities in comparison to concentrations in the media because an alternative route by using radioactive labeling would not reveal differences between compound classes and might also be compromised by a fraction of radioactivity that would be stored in insoluble fractions of the cell.

First, the relative proportion of peak intensity in the media was determined as percentage of the total peak intensity by

P_media _= [peak intensity_media _/peak intensity_(media +intracellular)_]

in order to give a perspective of how much of a compound might actually be found in the medium, be this by leakage or simply by excretion of this compound during cellular growth. Leakage was then defined as the difference of P_media _under quenching condition to those without quenching.

Leakage = P_media, quench _- P_media, no quench_

This calculation is also somewhat problematic because intracellular levels get altered when no quenching is employed due to continuing activity of intracellular metabolism and responses to the stress conditions during centrifugation. In many cases, however, this leads rather to an overestimation of leakage. For example, proline was systematically found at higher intracellular levels without quenching giving higher estimates for the 'relative leakage' which in fact was untrue. In the opposite, citrate was found at lower intracellular levels without quenching, giving actually rise to 'negative leakage' data. For such cases, we also compared the absolute difference in peak intensities in the media, to make sure potential leakages were not obscured by the differences in intracellular metabolism. In total, this method can be regarded as a conservative estimate of the number of peaks that were leaking into the medium due to compounds like proline which resulted in false overestimations of leakage due to continuing intracellular metabolism at 'no quenching' harvest conditions.

14 peaks were discarded because there were no significant differences between media and intracellular chromatograms, either because of peak artifacts, rapid exchange or due to chromatogram overloading (e.g. phosphate which was part of the medium). 218 peaks remained for the leakage comparison (table 1) of which the far majority of them (197 and 199, resp.) were found leaking at levels of less than 10% for both quenching methods. Only a few compounds were found at higher leakage levels, including putrescine for which its high intracellular concentration decreased at >30% under non-quenching conditions. This finding indicates high activity in putrescine metabolism in Chlamydomonas which in agreement with previous reports [28]. Even when taking this difference into account, P_media _were still higher under quenching conditions than under non-quenching conditions, revealing some losses of putrescine due to the methanol quenching solutions. For 26% methanol, 11% leakage was found but for 35% methanol, the leakage was increased to 18%, indicating that the Chlamydomonas cell walls might be weakened according to the higher concentration of methanol. Nevertheless, the overall observation as given in table 1 leads to the conclusion that for most compounds, leakage at the given methanol concentrations is below 5%. In addition, we present specific data for identified compounds under 35% methanol quenching buffer concentration in order to test if different classes of chemicals had different leakage rates [see Additional File 1]). We have not found a bias for any chemical class. For example, the level of cellular leaking of amino acids and sugars varied from well below 5% for most compounds to a little over 20% for a few outlier examples. Fatty acids showed particularly low variation in leakage of only 0–8.5% maximum. Leakage was also not determined by the relative cellular metabolite level, as estimated by the ion count intensity [see Additional File 1]. One of the most abundant metabolites, putrescine, was found at a high leakage rate of 18.6% but other abundant compounds such as glycerol-α-phosphate did not have detectable leakage. Taken together, these data confirm that cellular leakage indeed occurs at measurable quantities during quenching for some metabolites, but that most compounds remain unaffected by the procedure.

**Table 1:** Number of peaks that were found to be leaking at 26% and at 35% final methanol concentration in the quenching solution, calculated by the difference to peak intensities under non-quenching harvest conditions.

**Leakage level**	**< 5%**	**<10%**	**<15%**	**<20%**	**<25%**	**<30%**	**Total**
# of peaks leaking at 26% MeOH	164	25	15	8	4	2	218
# of peaks leaking at 35% MeOH	159	38	12	6	3	0	218

### Homogenization of cells performs better with steel ball than with glass bead milling

Before homogenization, cells were centrifuged and lyophilized at -50°C. The lyophilization step was introduced in addition to the benchmark protocol in order to store and accumulate samples for larger studies. Lyophilization eliminates any remaining interstitial water and thus efficiently inhibits any enzymatic reaction during storage. Additionally, residuals of the quenching solution are removed which enables parallel analysis of transcript and metabolite levels from the same sample, as it had been worked out for *Saccharomyces cerevisiae *[29].

Under the benchmark protocol, *Chlamydomonas *cells have to be disrupted by hand grinding with mortar and pestle in liquid nitrogen prior to extraction. Although this procedure certainly provides a valid homogenate of individual cells, it is certainly too laborious and consumes large quantities of liquid nitrogen when a high number of samples are to be prepared. Instead, two alternative methods were tested for efficiency of cell disruption and quantitative precision of metabolite profiles ('study 1'). One method involved glass beads for grinding in a mixer mill, similarly to protocols used in microbial preparations or minute quantities of plant tissues [30]. The other method employed a single 5 mm i.d. steel ball, similar to protocols that are successfully used for sample preparation of plant tissues [20]. In both comparisons, approximately 6 × 10^6 ^*Chlamydomonas *cells were used, equivalent to around 0.75 mg dry weight after lyophilization of cells. This low amount of material demanded that the extraction step was carried out concomitant with the homogenization in order to prevent losses due to adsorption on surfaces. Therefore, two different extraction mixtures were employed to test different recoveries of metabolites. In addition, great care had to be taken not to remove cellular debris in conjunction with taking out the supernatant extraction solvent. This proved especially difficult for the grinding method employing glass beads due to the large volume of glass beads. In order to achieve high extraction efficiency, two subsequent extraction steps were employed for both methods and the supernatant aliquots were combined. Extracts were dried and prepared for metabolite profiling as given in the methods section.

**Figure 3:**
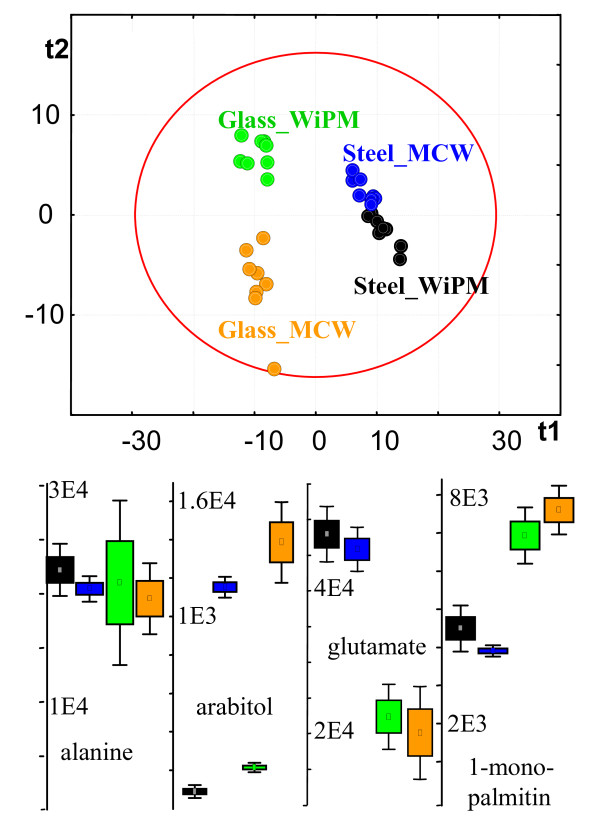
Study 1 on *C. reinhardtii *homogenization and extraction protocols, *N *= 9. Black: steel ball disruption + extraction by water/isopropanol/acetonitrile (WiPA). Blue: steel ball + methanol/chloroform/water (MCW). Green: glass bead disruption + WiPA. Orange: glass bead disruption + MCW. Upper panel: Supervised partial least square analysis (PLS); red circle: 3 × S.E. distance. Lower panel: Univariate one-way analysis of variance; units: normalized peak heights. Boxes show 1 × S.E, whiskers indicate 1.96 × S.E. data distribution.

**Figure 4:**
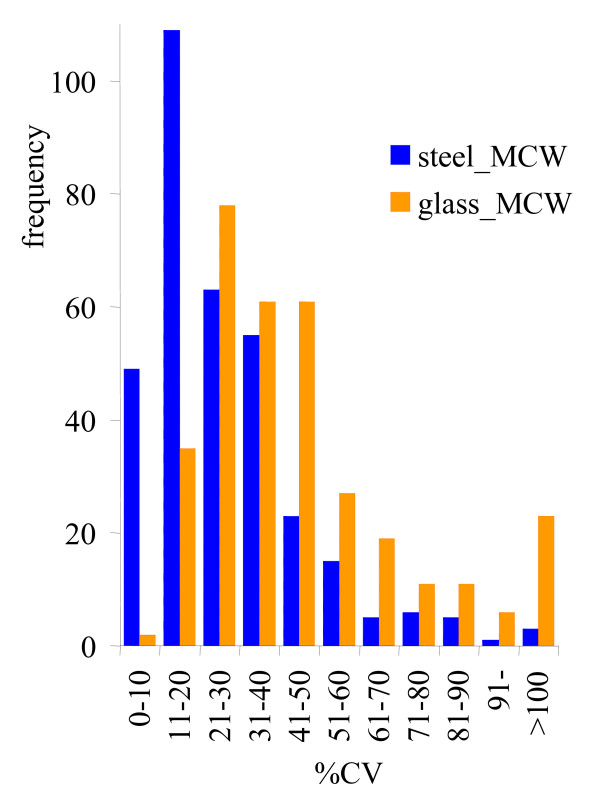
Frequency distribution of precision of *C. reinhardtii *metabolite profiling; *N *= 9; study 1. Blue: steel ball disruption + extraction by water/isopropanol/acetonitrile (WiPM). Orange: glass bead + methanol/chloroform/water (MCW). Both identified and unidentified metabolites were used as reported by the mass spectral database BinBase. CV = coefficient of variance.

A typical GC-TOF chromatogram consisted of about 750 peaks that were detectable at s/n 10 using the ChromaTOF vs. 2.25 software [31]. However, consistent and reliable metabolite profiling needs to ensure that all reported peaks fulfill high quality criteria with respect to mass spectral matching and retention index, and that each peak in a chromatogram can only be associated to a single metabolite. Common vendor software does not ensure this scrutiny. We have therefore reduced the number of reported peaks by employing a multi-tiered mass spectral annotation approach as outlined in the method section. 334 metabolite signals were determined to be consistent and reproducible peaks using the BinBase algorithm, of which 80 could be identified as non-redundant metabolites. Data can be downloaded at [32], detailing all detected peaks, identified metabolites, retention indices, mass spectra and quantification ions. In addition, raw chromatogram files can be downloaded to enable researcher comparisons of different data processing methods. Extraction efficiency of metabolites was estimated by comparing the sum intensity of all identified peaks. Three of the four methods gained similar overall peak intensities, except for the method employing grinding by glass beads and extraction with methanol/chloroform/water (MCW). Overall metabolite extraction efficiency by the glass bead/MCW method was 30–40% lower than by the other three methods (*p *= 0.00014) and it further comprised a clear outlier sample with even lower extraction efficiency. In order to enable comparison of relative extraction efficiency of individual compounds and to compare the overall metabolite profiles, data were normalized to the sum intensity of identified peaks of each chromatogram. Multivariate statistics demonstrated for both unsupervised principal components analysis (PCA) and supervised partial least square (PLS) analysis that the dominant factor separating metabolic profiles was associated with the method of grinding and homogenization rather than the two solvents that were employed. Only supervised PLS analysis could separate the two solvent systems (fig. 3) for each grinding method, but not unsupervised PCA. Univariate analysis of variance showed that a number of metabolites were equally well extracted by the four methods (e.g. alanine, fig. 3) whereas other compounds were either found at higher levels under metal ball grinding (e.g. glutamate, fig. 3) or at lower levels (e.g. 1-monopalmitin). Very few metabolites were only detectable by using one solvent system but not the other, specifically arabitol. The differential extraction efficiency of metabolites using the two different grinding systems could not easily be attributed to specific chemical classes. However, two considerations allowed a clear decision in favor of using steel ball homogenization: (a) sample handling by using steel balls is easier and therefore more reliable for high throughput applications and (b) the reproducibility of metabolite quantifications was best using steel ball grinding and methanol/chloroform/water (MCW) extraction (fig. 4 and table 2). Figure 4 exemplifies the frequency at which metabolites were quantified in 10% CV precision intervals for the steel ball/MCW method and the glass bead/MCW method. A high number of compounds had very high technical error rates using the glass bead/MCW method that resulted to a poor median precision of 30% CV for the identified metabolites and 39% over all 334 metabolite signals (table 2). The steel ball/MCW method provided good precision with a median of 16% CV for the identified compounds and 22% technical error over all metabolites (figure 4 and table 2). In comparison, extractions with water/isopropanol/methanol had intermediate precision but again, the steel ball grinding method proved to perform better than homogenizations by glass beads (table 2). Therefore, homogenization by steel ball milling was carried forward to study 2, investigating the impact of different extraction solvents.

**Table 2:** Technical error rates for metabolite profiling of *Chlamydomonas reinhardtii *cells.

**Homogenization**	**Solvent system**	**Identified metabolites**	**All peaks**
glass beads	MCW	30%	39%
glass beads	WiPM	26%	34%
steel ball	MCW study1	16%	22%
steel ball	MCW study2	18%	23%
steel ball	WiPM study1	21%	30%
steel ball	WiPM study2	25%	38%
steel ball	MeOH	30%	40%
steel ball	WiPA	28%	38%
steel ball	MCW (10/3/1)	30%	37%

**Figure 5:**
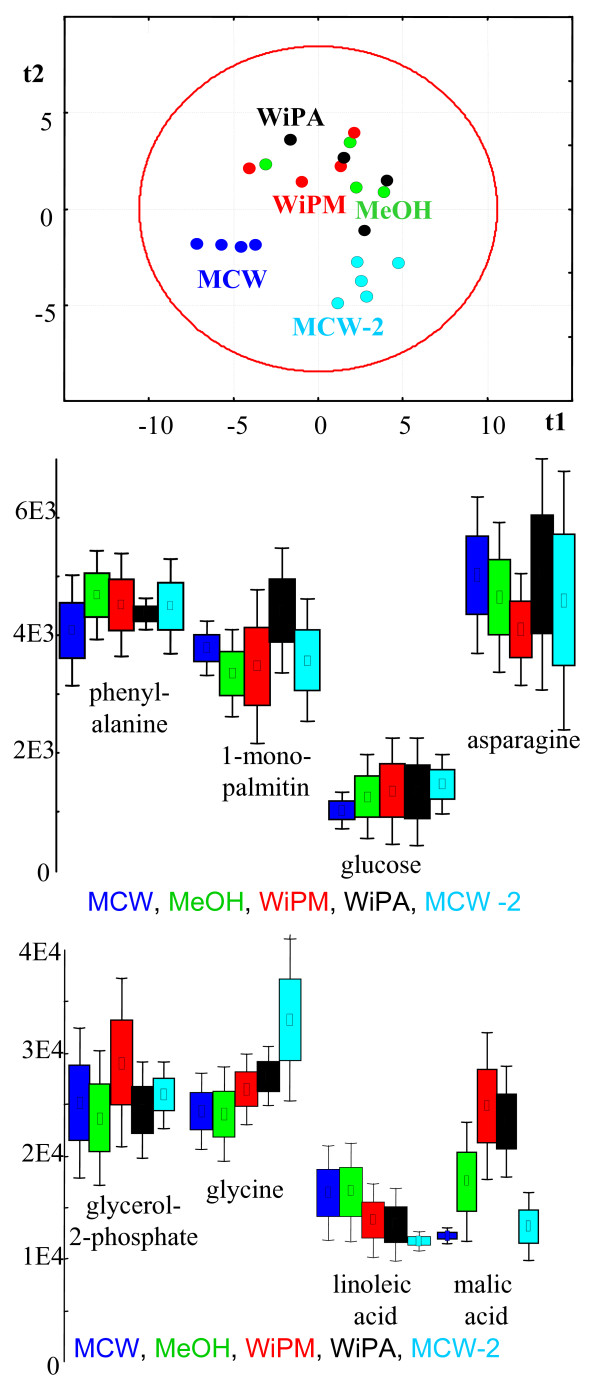
Study 2. Comparison of different extraction solvents for metabolite profiling after steel ball disruption of *C. reinhardtii *cells; *N *= 4–5. Blue: methanol/chloroform/water at 5/2/2 ratios (MCW); Green: 100% methanol; Red: water/isopronaol/methanol at 2/2/5 ratio (WiPM); Black: water/isopropanol/acetonitrile at 2/2/5 ratio (WiPA); Light blue: methanol/chloroform/water at 10/3/1 ratios (MCW-2) Upper panel gives a supervised partial least square analysis (PLS); Mid and lower panels show univariate one-way analysis of variance by box-whisker plots.

The median precision (as relative coefficient of variance) is given for the 80 compounds identified by BinBase, resp. over all 334 peaks that were annotated metabolic signals by BinBase. Study 1 comprised the comparison of grinding methods, study 2 compared different solvents. Abbreviation of solvents as given in figures 3–5.

### Different solvent systems have little impact on metabolite profiling in Chlamydomonas

Study 1 used showed a higher efficacy by using steel ball grinding compared to the classic glass bead disruption which can best be seen in table 2. A second study on method optimization was carried out utilizing five different systems for extraction solvents. Since it was intended to use even lower number of cells for biological studies, only 2.5 × 10^6 ^cells/ml were employed for comparing the impact of different solvent systems on the metabolite extraction efficiency. Therefore, studies 1 and 2 were not fully identical and results could not directly be plotted in a single multivariate graph whereas overall precisions can be compared using median data as given in table 2. Methanol/chloroform/water (MCW) was used at volume ratios 5/2/2 [20] as used in study 1 and also successfully employed in extraction of plant tissues. In addition, a second MCW mixture was used at volume ratios 10/3/1 (MCW-2) as reported before as benchmark method for metabolite profiling of Chlamydomonas cells. Reportedly, the relative amount of water was reduced in the MCW-2 mixture because the Chlamydomonas cells themselves comprised a high amount of water; however, we here tested lyophilized cells which reasonably required adding more water back to a 5/2/2 solvent ratio. Pure methanol was employed as comparison due to a report on successful application of this method for yeast metabolite profiling [15]. In order to remain as mild as possible, boiling extraction, acidic or alkaline conditions were not employed because these parameters tend to focus on specific chemical classes (e.g. acids or amines). Additionally, ternary mixtures of water, isopropanol and methanol (WiPM) and water, isopropanol and acetonitrile (WiPA) at solvent ratios 2/5/5 were tested because these solvent systems were demonstrated to be highly useful for human tissues and blood plasma [21]. Isopropanol is an excellent solvent for lipophilic constituents but it does not dissolve very nonpolar compounds such as waxes. Therefore, WiPA methods can be applied as a method for metabolome extraction excluding certain fractions. For example, LC/MS analysis of plant tissues is enabled by WiPA methods despite the potentially high wax contents in plants. Furthermore we have observed that any solvent mixture containing chloroform immediately enforces rapid denaturation and coagulation of cellular proteins. Depending on the sample matrix, this process may be helpful by establishing a complete stop of all enzymatic activity but it also might be detrimental by increasing the risk of co-precipitation of lipophilic metabolites and compounds that are closely associated with protein complexes.

Evaluation of relative performance of the five extraction systems was equivalent to study 1. Four of the five systems did not have significant differences on overall extraction efficiency of identified compounds except the benchmark method (MCW-2). This method yielded on average 27% lower raw peak intensities for the fraction of identified metabolites (*p *= 0.004) when compared to the MCW method with higher water contents. Free fatty acids and basic compounds were particularly more abundant in the MCW method than in the MCW-2 method; however, it cannot be excluded that this observation is a statistical artifact due to the low number of replicate samples utilized in study 2. Again, in order to compare the relative performance of the solvent systems, all data were normalized to the sum intensity of the identified metabolites for each sample. Unsupervised PCA analysis failed to separate the different systems (plot not shown), indicating that the overall metabolite profiles were highly similar. Univariate analysis of variance confirmed this notion (figure 5) as most compounds did not show statistically significant differences in any solvent. Only few compounds such as malate and citrate were found at lower proportions in the normalized data sets of the two MCW methods. These subtle differences were sufficient to discriminate both methods from the other three solvent systems by supervised PLS analysis (fig. 5). Importantly, the MCW method was again outperforming the overall precision of metabolite profiling with very median technical errors of 18% CV for the identified metabolites and 23% CV over all peaks reported by BinBase (table 2). No single chemical class of compounds was significantly different or even undetectable. We therefore confidently concluded that the parameter of solvent composition had little effect on composition and extraction efficiency of metabolites from Chlamydomonas. This effect can be explained by the high ratio of solvent amount to biological material of approximately 1700:1 (w/w) which was furthermore carried out in two sequential extraction steps. This ratio is around 50-fold higher than in other reports on metabolite profiling and hence might account for the lack of eminent differences between the solvent systems. Nevertheless, the method involving methanol/chloroform/water at solvent ratios 5/2/2 provided best analytical precision with very similar values in both method optimization studies. This high level of precision might be due to the effect of rapid enzyme inactivation as observed in other studies as well. We hence used the steel ball/MCW method for a proof-of-principle study on control of *Chlamydomonas *metabolism.

### Chlamydomonas reinhardtii cell cultures comprise drastically different metabolic phenotypes depending on time points of harvest

**Figure 6:**
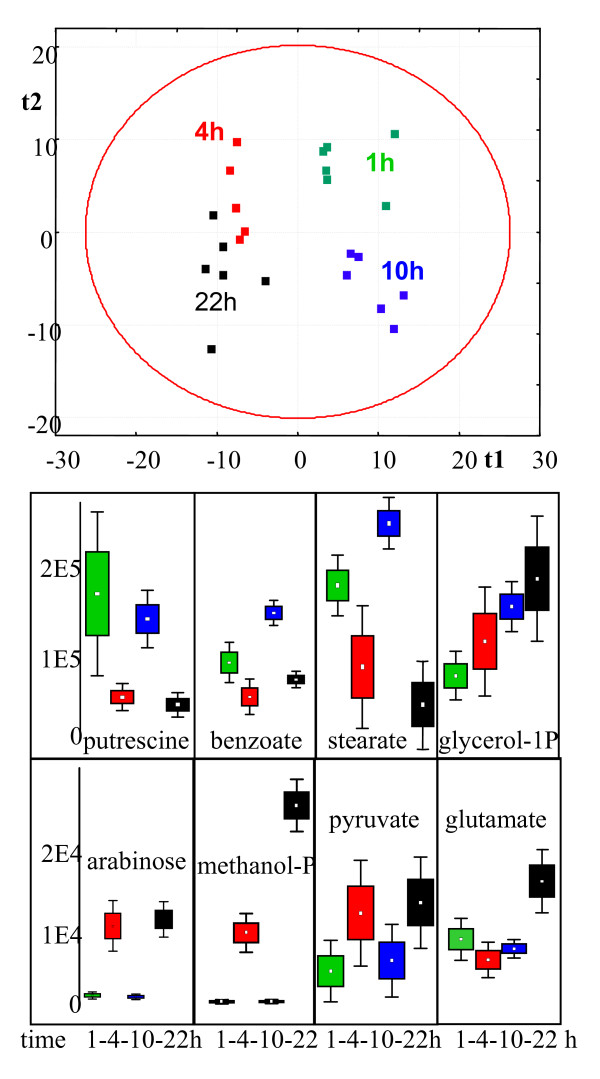
Study 3. Application of the optimized method for metabolite profiling of *C. reinhardtii *cells on different time points after inoculation of fresh cultures at 5 × 10^5 cells/mL ; *N *= 6. Green: 1 h, Red: 4 h, Blue: 10 h, Black: 22 h after inocculation. Upper panel: discrimination of metabolic phenotypes by partial least square analysis (PLS); Mid and lower panels: differential control of metabolism for selected compounds. One-way breakdown analysis of variance by box-whisker plots.

In an effort to establish a baseline of Chlamydomonas metabolism, four time points were selected subsequent to starting growth by inoculation with a starting culture of 2.5 × 10^6 ^cells. The underlying rational for selecting these time points was that it is known that (under optimal growth conditions), cell cycles of Chlamydomonas take about 5–8 h doubling times. By choosing four different time points, it was quite unlikely that all four time points would coincide in roughly identical cell cycles. Although cells were not synchronized, it can be assumed that in this short time frame most cells will undergo similar initial growth rates and consequently a similar number of cell divisions. Therefore, our hypothesis was that there is a large metabolic difference between the time points of harvest, especially between 4 and 10 h of growth because presumably at some time between these two harvests, most cells would duplicate. However, due to the stress conditions that might have been induced during transfer of cells and due to the initial lag time for starting the first cell division, this experimental design did not aim at testing specific regulatory points or metabolic oscillations. Indeed, both PCA (not shown) and PLS analysis confirmed this hypothesis (figure 6). In both multivariate analyses, vector 1 separated time points 1 h and 10 h from harvests at 4 h and 22 h. Moreover, PLS analysis clearly separated time points 1 h and 10 h by vector 2. Interestingly, samples at time point 1 h were found more homogeneous than at 4 h and 22 h, pointing to an increasing difference in the number of cells that are at slightly different phases of the cell cycles. PLS vector 3 was still significant by discriminating harvests at 4 h from 22 h (plot not shown). The overall metabolic differences were also apparent by univariate analysis of variance (fig 6) as demonstrated for selected metabolites. For example, putrescine, stearate and benzoate levels were found to be higher at 1 h and 10 h compared 4 h and 22 h,. A smaller number of compounds showed opposite patterns with higher levels at 4 h and 22 h than at 1 h and 10 h, e.g. arabinose and methanolphosphate. In a third category, compounds were significantly higher at 22 h than at other time points, such as for glycerol-1-phosphate or glutamate. However, the study design did not allow for more in-depth biochemical or physiological interpretations because too few time points were taken, the cells were not synchronized and might have been responding to additional stimuli during cell transfer. However, it becomes clear that any report on metabolic control in *Chlamydomonas reinhardtii *necessarily needs to state at which time point, cell density and cell cycle harvests were taken.

## Conclusion

Compared with proteins or nucleic acids, metabolite pools comprise more rapid molecular turnover and possess a wide variety of physicochemical properties. Therefore, methods need to be carefully developed and validated with respect to metabolome coverage and analytical precision. Taking all our evaluation criteria into account, we cautiously conclude that cell homogenization by steel ball milling concomitant with methanol:chloroform:water extraction (5:2:2) is best suited for *Chlamydomonas *metabolite profiling. The optimized method presented here for metabolite profiling of *Chlamydomonas *reinhardtii enables rapid analysis of a high number of replicate samples with considerably lower efforts than the previously published benchmark method. Technical errors were lower than reported for most other systems, including plant tissues. We could prove that *Chlamydomonas *cultures undergo drastic changes in overall metabolic levels depending on the duration of growth and the number of cell cycles. This work lays the ground to more in depth studies of biochemical networks in this model organism.

## Authors' contributions

DYL carried out the experimental work, initiated the statistical evaluations and drafted the manuscript. OF conceived the study, devised the experimental design, performed statistical analyses, improved the graphics and revised the manuscript. All authors read and approved the final manuscript.

## Supplementary Material

Additional file 1 Metabolite specific leakage rates. The data detail the leakage rates at 35% methanol concentration in the quenching solution, compared to metabolite concentrations that were analyzed without cellular quenching. Click here for file

## References

[B1] Hell R, Leustek T (2005). Sulfur metabolism in plants and algae – a case study for an integrative scientific approach. Photosynthesis Research.

[B2] Lohr M, Im CS, Grossman AR (2005). Genome-based examination of chlorophyll and carotenoid biosynthesis in Chlamydomonas reinhardtii. Plant Physiology.

[B3] Jain M, Shrager J, Harris EH, Halbrook R, Grossman AR, Hauser C, Vallon O (2007). EST assembly supported by a draft genome sequence: an analysis of the Chlamydomonas reinhardtii transcriptome. Nucleic Acids Research.

[B4] Gutman BL, Niyogi KK (2004). Chlamydomonas and Arabidopsis. A dynamic duo. Plant Physiology.

[B5] Gonzalez-Ballester D, de Montaigu A, Higuera JJ, Galvan A, Fernandez E (2005). Functional genomics of the regulation of the nitrate assimilation pathway in Chlamydomonas. Plant Physiology.

[B6] Lilly JW, Maul JE, Stern DB (2002). The Chlamydomonas reinhardtii organellar genomes respond transcriptionally and post-transcriptionally to abiotic stimuli. Plant Cell.

[B7] Bolling C, Fiehn O (2005). Metabolite profiling of Chlamydomonas reinhardtii under nutrient deprivation. Plant Physiology.

[B8] de Koning W vDK (1992). A method for the determination of changes of glycolytic metabolites in yeast on a subsecond time scale using extraction at neutral pH. Analytical biochemistry.

[B9] Wittmann C, Kromer JO, Kiefer P, Binz T, Heinzle E (2004). Impact of the cold shock phenomenon on quantification of intracellular metabolites in bacteria. Analytical Biochemistry.

[B10] Bolten CJ, Kiefer P, Letisse F, Portais JC, Wittmann C (2007). Sampling for Metabolome Analysis of Microorganisms. Analytical Chemistry.

[B11] Jaki BU, Franzblau SG, Cho SH, Pauli GF (2006). Development of an extraction method for mycobacterial metabolome analysis. Journal of Pharmaceutical and Biomedical Analysis.

[B12] Gullberg J, Jonsson P, Nordstrom A, Sjostrom M, Moritz T (2004). Design of experiments: an efficient strategy to identify factors influencing extraction and derivatization of Arabidopsis thaliana samples in metabolomic studies with gas chromatography/mass spectrometry. Analytical Biochemistry.

[B13] Schaub J, Schiesling C, Reuss M, Dauner M (2006). Integrated Sampling Procedure for Metabolome Analysis. Biotechnology Progress.

[B14] Maharjan RP, Ferenci T (2003). Global metabolite analysis: the influence of extraction methodology on metabolome profiles of Escherichia coli. Analytical Biochemistry.

[B15] Villas-Boas SG, Hojer-Pedersen J, Akesson M, Smedsgaard J, Nielsen J (2005). Global metabolite analysis of yeast: evaluation of sample preparation methods. Yeast.

[B16] Jiye A, Trygg J, Gullberg J, Johansson AI, Jonsson P, Antti H, Marklund SL, Moritz T (2005). Extraction and GC/MS analysis of the human blood plasma metabolome. Analytical Chemistry.

[B17] Harris E (1998). The Chlamydomonas Sourcebook:. A Comprehensive Guide to Biology and Laboratory Use.

[B18] Crutchfield A, Diller K, Brand J (1999). Cryopreservation of Chlamydomonas reinhardtii (Chlorophyta). European Journal of Phycology.

[B19] Farr TJ, Huppe HC, Turpin DH (1994). Coordination of Chloroplastic Metabolism in N-Limited Chlamydomonas-Reinhardtii by Redox Modulation .1. The Activation of Phosphoribulosekinase and Glucose-6-Phosphate-Dehydrogenase Is Relative to the Photosynthetic Supply of Electrons. Plant Physiology.

[B20] Weckwerth W, Wenzel K, Fiehn O (2004). Process for the integrated extraction identification, and quantification of metabolites, proteins and RNA to reveal their co-regulation in biochemical networks. Proteomics.

[B21] Kind T, Fiehn O, Weckwerth W, Totowa NJ (2006). Metabolite profiling in blood plasma. Metabolomics: Methods and Protocols.

[B22] Fiehn O, Wohlgemuth G, Scholz M (2005). Setup and Annotation of Metabolomic Experiments by Integrating Biological and Mass Spectrometric Metadata. Proc Lect Notes Bioinformatics.

[B23] Scholz M, Fiehn O (2007). SetupX – a public study design database for metabolomic projects. Pacific Symposium on Biocomputing.

[B24] Katajamaa M, Miettinen J, Oresic M (2006). MZmine: toolbox for processing and visualization of mass spectrometry based molecular profile data. Bioinformatics.

[B25] Denkert C, Budczies J, Kind T, Weichert W, Tablack P, Sehouli J, Niesporek S, Konsgen D, Dietel M, Fiehn O (2006). Mass spectrometry-based metabolic profiling reveals different metabolite patterns in invasive ovarian carcinomas and ovarian borderline tumors. Cancer Research.

[B26] Giroud C, Gerber A, Eichenberger W (1988). Lipids of Chlamydomonas-Reinhardtii – Analysis of Molecular-Species and Intracellular Site(S) of Biosynthesis. Plant and Cell Physiology.

[B27] Koek MM, Muilwijk B, Werf MJ van der, Hankemeier T (2006). Microbial metabolomics with gas chromatography/mass spectrometry. Analytical Chemistry.

[B28] Theiss C, Bohley P, Voigt J (2002). Regulation by Polyamines of Ornithine Decarboxylase Activity and Cell Division in the Unicellular Green Alga Chlamydomonas reinhardtii. Plant Physiology.

[B29] Martins AM, Sha W, Evans C, Martino-Catt S, Mendes P, Shulaev V (2007). Comparison of sampling techniques for parallel analysis of transcript and metabolite levels in Saccharomyces cerevisiae. Yeast.

[B30] Portillo M, Fenoll C, Escobar C (2006). Evaluation of different RNA extraction methods for small quantities of plant tissue: Combined effects of reagent type and homogenization procedure on RNA quality-integrity and yield. Physiologia Plantarum.

[B31] Weckwerth W, Loureiro ME, Wenzel K, Fiehn O (2004). Differential metabolic networks unravel the effects of silent plant phenotypes. Proceedings of the National Academy of Sciences of the United States of America.

[B32] SetupX – Study Design Database. Public repository of metabolomics data at the UC Davis Genome Center. http://fiehnlab.ucdavis.edu:8080/m1/main_public.jsp.

