# Supplementary material for: High quality metabolomic data for Chlamydomonas reinhardtii
Source: Plant Methods. 2008 Apr 28;4:7. doi: 10.1186/1746-4811-4-7 (PMC2377246; doi:10.1186/1746-4811-4-7)
Supplement: Additional file 1 — Metabolite specific leakage rates. The data detail the leakage rates at 35% methanol concentration in the quenching solution, compared to metabolite concentrations that were analyzed without cellular quenching. [file 1746-4811-4-7-S1.pdf]

Metabolite	Cell leakage	Signal intensity
1,2,4-benzenetriol	6.2%	14997
1-hexadecanol	<5%	7509
1-monostearin	<5%	8510
2-isopropylmalic acid	<5%	11177
2-monopalmitin	<5%	4535
3-phosphoglycerate	<5%	164233
5'-deoxy-5'-methylthioadenosine	<5%	11898
adenine	<5%	25719
adenosine	<5%	15094
adenosine-5-monophosphate	<5%	31175
alanine	<5%	20549
α-ketoglutaric acid	<5%	18884
arabinose	<5%	8765
arginine + ornithine	14.5%	24534
asparagine	<5%	6960
aspartic acid	6.9%	22632
beta alanine	<5%	37329
capric acid	<5%	88763
cellobiose	<5%	1944
cholesterol	5.4%	10804
citric acid	<5%	29347
cytidine-5'-diphosphate deriv.	<5%	60109
dehydroascorbate	7.5%	3368
elaidic acid	<5%	31011
fructose	6.4%	2152
fructose-6-phosphate	8.8%	3628
fumaric acid	<5%	74735
galactose	15.9%	12410
galactose-6-phosphate	9.8%	1289
glucose-6-phosphate	7.1%	6817
glutamic acid	9.7%	47472
glyceric acid	<5%	14250
glycerol	9.9%	148326
glycerol-α-phosphate	<5%	1150504
glycerol-β-phosphate	<5%	32132
glycine	8.8%	222323
guanine	<5%	16517
guanosine	<5%	786850
heptadecanoic acid	5.3%	25013
icosenoic acid	<5%	18040
inositol myo-	13.7%	14571

Metabolite	Cell leakage	Signal intensity
inositol-4-monophosphate	<5%	139384
isoleucine	16.9%	47759
lauric acid	5.1%	153882
leucrose	<5%	15011
linoleic acid	<5%	35074
linolenic acid	<5%	296829
lysine	<5%	220806
malate	7.1%	76200
maltotriose	<5%	47154
methionine	<5%	10102
methionine sulfoxide	5.1%	6541
1-monopalmitin	<5%	10606
myristic acid	5.8%	28001
octadecanol	<5%	6740
oleic acid	<5%	26038
palmitic acid	<5%	395453
palmitoleic acid	8.3%	39788
pantothenic acid	<5%	5168
pelargonic acid	<5%	194072
pentadecanoic acid	8.5%	15358
phenylalanine	11.5%	34765
phytol	<5%	754448
previtamin D3	<5%	46128
proline	24.1%	35284
pseudo uridine	6.8%	8629
putrescine	18.6%	1227790
ribose-5-phosphate	<5%	1029
serine	11.7%	165431
stearic acid	<5%	871127
sucrose	21.6%	4002
threitol	<5%	5316
threonine	9.7%	67334
tocopherol	<5%	42873
trehalose	14.2%	2204
tyrosine	7.1%	38841
urea	<5%	59696
xylitol	5.7%	3871

Metabolite specific cellular leakage rates. These rates were determined when using quenching solvents containing 35% methanol (v/v) in comparison to media blanks and media after quenching. Signal intensities in counts-per-second of the time-of-flight mass spectrometer are given using the quantification ions as in the annotation result downloads at [32].
